# Occurrence and severity of spontaneous exposure of cover screw after dental implant placement

**DOI:** 10.34172/japid.2021.017

**Published:** 2021-11-24

**Authors:** Ramin Negahdari, Arezou Ghoreishizadeh, Mohammad Ali Ghavimi, Atefeh Soltanpour, Sepideh Bohlouli

**Affiliations:** ^1^Department of Prosthodontics, Faculty of Dentistry, Tabriz University of Medical Sciences, Tabriz, Iran; ^2^Department of Pediatric Dentistry, Faculty of Dentistry, Tabriz University of Medical Sciences, Tabriz, Iran; ^3^Department of Oral and Maxillofacial Surgery, Faculty of Dentistry, Tabriz University of Medical Sciences, Tabriz, Iran; ^4^Department of Oral Medicine, Faculty of Dentistry, Tabriz University of Medical Sciences, Tabriz, Iran

**Keywords:** Implant, Implant screw, maxillary premolars, osseointegration

## Abstract

**Background:**

Perforation of the soft tissues overlying the dental implant, resulting in early and spontaneous exposure of cover screws between stages I and II of the two-staged implant placement procedure, is a common problem that can disrupt the primary repair and osseointegration process. The present study aimed to investigate the prevalence of spontaneous exposure of cover screws in dental implants and identify the related risk factors.

**Methods:**

The present retrospective, descriptive-analytical study enrolled 40 patients with 182 dental implants in the second stage of the implant placement procedure. Data on patient-related and implant-related classified variables were collected, and all the samples were examined for cover screw exposure based on the classification by Tal. First, the overall prevalence of cover screw exposure was calculated. Then, statistical analysis was performed using SPSS 24 to investigate the effect of different variables on this exposure. The chi-squared test was used at the bivariate level, while the logistic regression was used at the multivariate level.

**Results:**

Of 40 participants with 182 implants, 17 implants (9.3%) in 9 patients (22.5%) became exposed to the oral cavity. In terms of severity, Class I exposure was the most common with seven implants. Moreover, Class III was the least common with only one implant. Using the logistic regression analysis, we found significant relationships between the dental implant exposure and the variables of overlying mucosal thickness (OR=24.7, P≤0.001), the duration between tooth extraction and implant placement (OR=9.6, P=0.005), and implant location in the jaw (OR=3.8, P=0.033). Moreover, exposure was more common in the maxillary premolar area (22.5%) than in other locations. Also, there was a significant relationship between implant exposure and lateral augmentation (OR=0.20, P=0.044), indicating the higher risk of exposure in implants with lateral augmentation than those without augmentation.

**Conclusion:**

Despite the limitations of this retrospective study, its results showed that three factors, including the overlying mucosal thickness of <2 mm, implant placement in fresh extraction sockets, and maxillary implants, especially at the location of maxillary premolars, were strong predictors of spontaneous implant exposure.

## Introduction

In the last two decades, extensive research and remarkable advances in dental implant technology have led to the introduction of the missing tooth replacement treatment using intraosseous implants as a standard and long-term therapeutic modality.^1^ Dental implant placement is performed using the single-stage and two-stage methods, both showing similar results in the dimensions and thickness of soft and hard tissues surrounding the implant.^2,3^ However, the two-staged method is more common. This technique was introduced by Branemark et al in 1969 and led to successful results.^4^ In this method, during phase I (osseointegration stage), the implant is buried under the gingival tissue and isolated from the dental environment to provide suitable sterile conditions without stress and trauma for primary repair.^5^ However, perforation of the gingival tissue overlying the implant between phases I and II is common, leading to early and spontaneous exposure of the cover screws^6^ with a prevalence of 6%^7^ to 13.7%^8^ in various studies. If left untreated, the gingiva-implant interface can be a suitable location for microbial plaque accumulation and colonization of opportunistic bacteria, such as Prevotella, β-hemolytic streptococci, and Fusobacterium, resulting in the inflammation of the subepithelial connective tissue, crestal bone resorption, and eventually, osseointegration impairment.^8-10^

Various studies have reported several risk factors for early crestal bone resorption as follows:^10-12^ intraoperative problems, insufficient size of bone, insufficient initial stability of the implant, lack of keratinized gingiva, application of early forces to implant before osseointegration due to implant micromovements, hazardous behaviors of patients such as smoking, and systemic factors such as genetics or metabolic disorders.

However, only two studies have focused on the risk factors of spontaneous cover screw exposure in implants. According to Hertal et al,^13^ males, supracrestal implant placement, implant placement in the posterior jaws, and implants with platform-matching cover screws have a significantly increased rate of cover screw exposure. Moreover, Mendoza et al^19^ showed significant relationships between the spontaneous implant exposure and three factors of keratinized gingiva, implant placement method (fresh extraction socket or edentulous ridge), and use of GTR with bone grafts.

According to several studies, spontaneous cover screw exposure is directly related to the early crestal bone resorption and can aggravate the process of supporting bone resorption.^14-16^ If the diagnosis is delayed, the risk of bone loss increases. However, the gingival perforation diagnosis is often delayed because this problem can be asymptomatic and might be ignored by the patient. Therefore, early detection of cover screw exposure is vital to prevent further complications and minimize the risk of bone resorption by proper therapeutic interventions. For early diagnosis, the patient should be under regular follow-ups between phases I and II of implant placement, and high-risk patients should be recalled for more visits. The present study aimed to investigate the prevalence of spontaneous cover screw exposure in implants and identify the related risk factors.

## Methods

### Study population, inclusion, and exclusion criteria

The present retrospective, descriptive-analytical study used the census method for sampling. The study population included all the patients who underwent implant placement procedures in the implant ward of the Faculty of Dentistry, Tabriz University of Medical Sciences, during 2019-2020. The patients were screened based on the following eligibility criteria. Forty patients, including 18 females and 22 males, were considered eligible based on the inclusion and exclusion criteria and included in the study after signing informed consent forms.

### Inclusion criteria

Patients with available dental records and CBCT images taken from the implant area

Patients with a two-staged implant placement technique, who had only undergone the first stage

Use of DIO Implants (Busan, Korea)

Use of crestal pocket flap with or without a release and sutures using the 3-0 silk thread

The implant shoulder placed at the level of the bone crest

The second surgery to be performed 3-6 months following the first surgery

Favorable oral hygiene in the examination visit (free of plaque, active periodontal pocket, a plaque index of <50%).

### Exclusion criteria

1. Any mucosal disease, such as pemphigus vulgaris, lichen planus, erythema multiforme, and others
2. History of opioid and alcohol use
3. History of head and neck radiotherapy
4. History of temporary dental prosthesis use
5. Uncontrolled metabolic diseases, debilitating diseases, and pregnancy
6. Any symptom suggestive of implant placement failure, such as implant mobility, abscess, or active fistula
7. Vertical augmentation at the implant site

### The first stage of implant placement

The first stage of surgery was performed for all the patients by the maxillofacial surgery residents under the supervision of professors in the implant ward. Each patient received at least one implant (DIO Implant Systems, Busan, Korea). The implant site was exposed using the crestal pocket flap with or without release. The implant fixture placement was at the bone level. The patients needing lateral augmentation of the alveolar ridge or sinus lift and those undergoing fresh extraction socket implants with a distance longer than 2 mm between the implant surface and the socket wall underwent bone grafting. The bone graft was used in case of need for membranes. Finally, the flap was sutured using the 3-0 silk sutures (Hu-Friedy Mfg Company, Chicago, USA). All the patients were administered 1 gr of amoxicillin one hour before the surgery. The antibiotic administration was continued for 10 days in those with bone grafts. Moreover, chlorhexidine mouthwash (2%) was administered for all the patients for one week after surgery.

### Severity classification of cover screw exposure

In the second stage of implant placement (implant uncovering), all the 182 implants underwent thorough clinical examination by a maxillofacial surgeon.

### Exposure severity

There are different classes for cover screw exposure of implants (Table 1).

**Table 1:** The different classes for cover screw exposure of implants

**Class**	**Description**
**Class 0**	The entire cover screw of the implant is covered by the healthy mucosal soft tissue.^6^
**Class I**	There is a cleft in the mucosa overlying the implant that can be detected using a periodontal probe, but the cover screw surface cannot be observed without mechanical intervention.^8^
**Class II**	The overlying mucosa is perforated, and the cover screw is visible, but perforation borders do not overlap with cover screw borders at any point.^8^
**Class III**	The overlying mucosa is perforated, and the cover screw is visible, and perforation borders overlap with cover screw borders at some points.^6,8^
**Class IV**	The cover screw is completely exposed to the oral environment. The whole cover screw is visible.^8^

### Study variables and the related assessment

The following variables in two groups of patient-related and implant-related variables were extracted from the patients’ records and confirmed using self-reporting questionnaires or clinical examination. The data were recorded in a data collection form.

### Patient-related variables

1. Gender (male/female)
2. Age (three age groups: 18-39, 40-59, and ≥60)
3. Presence of uncontrolled systemic diseases, such as hypertension, diabetes, hypothyroidism, epilepsy, and others
4. Smoking (yes/no)

### Implant-related variables

1. Implant diameter (regular/narrow)

Narrow: implants with a diameter of 3 and 3.3 mm

Regular: implants with a diameter of ≥3.8

2. Implant length (short/long)

Short: implants with a length of 7-8.5 mm

Long: implants with a length of 10-14 mm

3. Keratinized gingival width (<2 mm and ≥2 mm): It was measured in the buccal part using a periodontal probe with an accuracy of 0.5 mm

4. The thickness of overlying mucosa (<2 mm ≥2 mm): It was measured after crestal incision and before the flap retraction using a #30 endo file with the file tip resting on the implant surface and the file stop resting on the gingiva

5. Location (anterior maxilla, posterior maxilla, anterior mandible, posterior mandible)

Anterior: implants at the location of incisors and canines

Posterior: implants at the location of premolars and molars

6. Type of edentulism (single-tooth edentulism, partial edentulism, complete edentulism of a quadrant)

7. The duration between tooth extraction and implant placement (fresh socket/delayed socket)

Fresh socket: <24 hours between tooth extraction and implant placement

Delayed socket: >24 hours between tooth extraction and implant placement

8. Quality of supporting bone (D1, D2, D3, D4, D5): It is classified into four groups based on the Hounsfield value in the CT scan

D1: >1250

D2: 850-1250

D3: 350-850

D4: 150-350

D5: <150

9. Augmentation (lateral augmentation, without augmentation) Only lateral augmentations were included in the study.

### Statistical analysis

Statistical analysis was performed in two steps of descriptive analysis and analytical analysis. In the descriptive analysis, descriptive indicators and frequency tables were used to describe the data. In the analytical statistics, the effect of each variable on the spontaneous cover screw exposure was individually investigated using the chi-squared test. Then, the variables with a P-value<0.2 in the bivariate chi-squared test were included in the multivariate analysis using the forward stepwise logistic regression for final investigation and calculation of odds ratio with a confidence interval of 95%. The significance level was considered 0.05, and the data analysis was performed using the SPSS 24.

## Result

### Spontaneous cover screw exposure

Of a total of 40 participants with 182 implants, nine patients (22.5%), including six men and three women, had spontaneous cover screw in at least one of their implants. Three patients experienced this in one implant, four had this problem in two implants, and two had exposures in three of their implants. Thus, the total number of exposed implants was 17 (9.3%). Therefore, each patient with cover screw exposure had two exposed implants on average.

**Figure 1:**
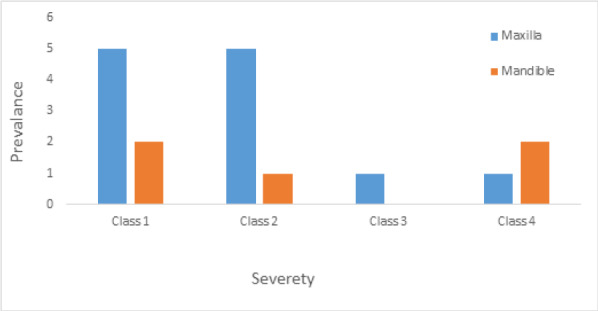
Frequency distribution and severity of exposed cover screws of implants placed in the maxilla and mandible in the study.

**Figure 2:**
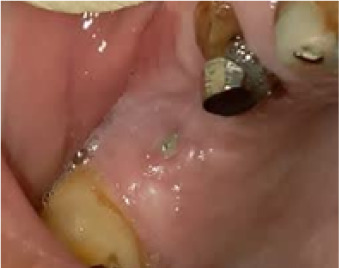
Class I exposure of a maxillary premolar implant.

**Figure 3:**
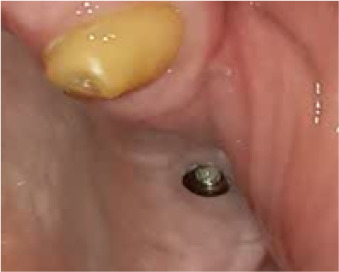
Class III exposure of a maxillary premolar implant.

**Figure 4:**
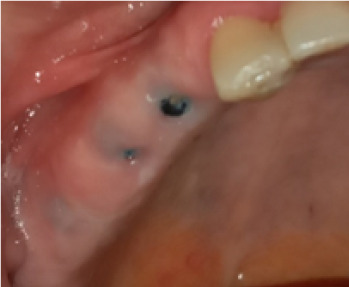
Class I exposure of a maxillary first premolar implant and Class II exposure of a maxillary canine implant.

**Figure 5:**
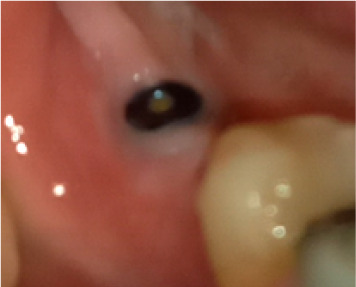
Class IV exposure of a mandibular first premolar implant.

**Figure 6:**
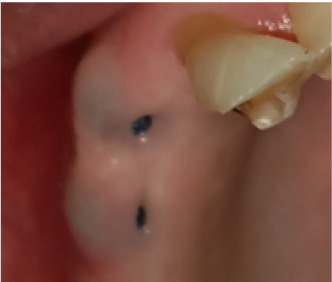
Class II exposure of a maxillary first premolar implant and a canine implant.

In terms of severity, Class I exposure was the most common with seven implants. Moreover, Class III was the least common with only one implant. The number of exposed maxillary and mandibular implants is presented in Figure 1. Also, Figures 2-6 show the exposure of maxillary premolar implants.

### Analytical analysis

No significant correlation was found between the outcome variable (exposure of at least one implant in each patient) and any of the patient-related variables (P≥0.05) (Table 2). It is worth mentioning that the smoking variable was eliminated because none of the patients were smokers.

**Table 2:** The correlations between patient-related variables and spontaneous cover screw exposure (patient-level assessments)

**Variable**	**Exposed**	**Unexposed**	**P-value**
**Age**			0.430
**Gender**			0.424
**Female**	3 (16.7%)	16 (72.7%)	
**Male**	6 (27.3%)	15 (83.3%)	
**Systemic Disease**			
**Hypertension**	1 (20%)	4 (80%)	0.886
**Hypothyroidism**	1 (25%)	3 (75%)	0.900
**Diabetes**	1 (50%)	1 (50%)	0.339
**Seizure**	1 (50%)	1 (50%)	0.339
**Others**	0	4 (100%)	0.256

The frequency percentages mentioned in parentheses were calculated individually for each row.

## Discussion

The spontaneous cover screw exposure of implants can be investigated in four aspects: histology, microbiology, etiology, and related consequences. Tal^6,17^ evaluated the histology and pathohistology of unexposed and exposed cover screws for the first time in two studies. Moreover, the microbiology of exposed cover screws was studied by Barboza et al.^18^ One of the potential consequences of this phenomenon, reported by Tal^8^ in his first study, was the impaired osseointegration and marginal bone resorption. These complications were investigated in several subsequent studies, all of which confirmed the higher marginal bone resorption in implants with partial exposure compared to unexposed implants. Therefore, there is almost no need for further studies. However, limited studies are available on the related etiology and risk factors, which are sometimes controversial. For example, Toljanic et al^10^ even reported that they could not identify the related etiology. Therefore, our primary objective was to investigate the prevalence of spontaneous implant exposure, while our secondary objective was to investigate the related risk factors. The present study showed prevalence rates of 22.5% and 9.3% for patients with spontaneous exposure and the implants exposed, respectively, consistent with previous studies (4.6-13.7%). However, Mendoza et al^19^ reported a prevalence of 63% for this problem, which was dramatically different from other studies. Moreover, they believed that this high prevalence could be due to the quality of suturing, flap tension, releasing flaps, and natural contraction of the flaps during the healing process.^19^ Moreover, the present study showed that seven implants had Class I exposure based on the exposure severity classification by Tal, while only one implant had Class III exposure. Therefore, Class I was the most common exposure type, while Class III was the least common. These findings were compatible with the study by Tal in the most common exposure type (Class I), while they were incompatible with the least common exposure type, which was reported as Class 4 in the study by Tal.^8^

In the present study, the outcome variable was the spontaneous cover screw exposure, while the dependent variables were the potential risk factors in two groups of patient-related variables (age, gender, smoking, and systemic diseases) and implant-related variables (width and thickness of the keratinized gingiva, bone density, duration from tooth extraction to implant placement, implant augmentation type, and implant location, length, and diameter). Other confounding variables, including implant system type, flap type, the sutures used, the distance between the bone crest and the implant shoulder at the time of placement, and the use of temporary prosthesis, were matched as much as possible. The smoking variable was eliminated because smoking was considered one of the exclusion criteria. The patient-level analysis did not show any significant correlation between the outcome variable and any patient-related variable. Moreover, the patient-related variables were also compared between the genders to rule out the effect of gender, and no significant inter-gender difference was found (P<0.05).

A study by Hertal et al^13^ investigated the effect of patient-related variables, including various systemic diseases, on spontaneous implant exposure and reported a significantly higher rate of implant exposure only in men. Moreover, a previous study showed a significant reduction of new bone formation and bone density in the cortical area around dental implants in an animal model with hypothyroidism.^20^ Also, it was reported that patients under treatment for hypothyroidism had a higher rate of soft tissue problems following implant placement.^21^ However, in the present study, only four patients were affected by hypothyroidism. Therefore, it was not possible to investigate the relationship between hypothyroidism and spontaneous implant exposure. Some previous meta-analyses and systematic reviews have shown a significant increase in the resorption of peri-implant marginal bone in diabetic patients compared to non-diabetic individuals.^22^ However, it is worth mentioning that only 5% of our participants were affected by diabetes. Therefore, it was not possible to investigate the relationship between diabetes and spontaneous implant exposure.

Concerning implant-related variables, the bivariate analysis using the chi-squared test and the multivariate analysis using the logistic regression showed a significant and positive correlation between spontaneous implant exposure and the following implant-related variables: overlying mucosal thickness of <2 mm, fresh socket placement, and location of the implant, especially at the location of maxillary premolars. In fact, compared to previous studies, our innovation aimed to investigate the overlying mucosal thickness combined with other variables because no study has ever investigated the effect of this variable on the spontaneous cover screw exposure. Moreover, previous studies have acknowledged that not considering the soft tissue conditions might lead to incomplete conclusions and recommended further studies on the variables related to soft tissues.^13^

Mendoza et al^19^ investigated the effect of the width of the keratinized gingiva, GTR, and fresh socket implant placement on the spontaneous implant exposure and found no significant relationship. The present study was compatible with the mentioned study because we found no significant relationships between spontaneous implant exposure and the factors of the width of the overlying mucosa and the type of augmentation. However, we found that fresh socket implant placement had significant effects, which is different from the study above. Several studies have demonstrated the importance of sufficient keratinized gingiva around the implant after loading and prosthesis placement. Moreover, some studies have reported excessive plaque accumulation and further resorption of pre-implant soft tissue in the absence of keratinized gingiva.^23^ However, the present study showed that this variable was not a risk factor before the prosthesis placement when the implant is buried under the gingiva and that the thickness of the mucosa is more important than its width. It seems logical because thick tissue has a higher resistance against exposure than thin periodontal issues. However, Baqain et al^24^ showed that a lack of keratinized gingiva could be a strong predictor of early failure of implant treatment before the loading stage. Therefore, there is a need for further studies to refute or confirm these findings, and it is recommended that maximum effort be made to preserve the existing keratinized mucosa for the subsequent success of the implant treatment.

In the present study, the duration between tooth extraction and implant placement was significantly correlated with implant exposure. A double-blind study by Cavit et al^14^ also showed results compatible with ours. They showed that immediately placed implants (the mean duration between tooth extraction and implant placement was 40 days in this study) had a higher chance of implant exposure than those with delayed placement. However, a study on 124 patients with 493 implants showed that the likelihood of implant exposure was significantly higher in implants with a duration of more than three months between tooth extraction and implant placement,^25^ which was not compatible with the results reported by Cavit et al.^14^ Finally, two subsequent studies did not find a relationship between this variable and implant exposure.^13,19^ This controversy can be further investigated in future studies.

The present study did not show an increased rate of implant exposure in implants with lateral augmentation. The regression analysis showed that implants with lateral augmentation had a lower chance of exposure than those without augmentation. Two other studies also did not find a significant relationship between augmentation and implant exposure.^13,19^ Moreover, another study reported no exposure at the locations using grafts from membranes.^10^

Regarding the implant location, we observed a higher likelihood of exposure in the maxillary implants, especially those at the posterior maxilla and the maxillary premolar area. However, in the bivariate analysis between anterior and posterior areas, no significant difference was observed because the implants at the location of maxillary molars had a lower rate of exposure, which could be due to the higher thickness of the overlying mucosa. Moreover, no exposure was observed in the anterior maxilla. Therefore, despite the higher prevalence of exposure in the maxilla than the mandible, no significant difference was found between the jaws in the bivariate analysis. Thus, we performed a bivariate analysis for dental locations to eliminate this difference in prevalence and found a significant difference between the maxillary premolar area and other dental locations in the exposure rate. This finding was more detailed than the findings by Hertal et al^13^ on the higher rate of exposure in the posterior areas of the jaws. Finally, logistic regression analysis showed a higher chance of exposure in the maxilla than in the mandible. No exposure was observed in implants placed in single-tooth locations, which seemed reasonable due to the protective effect of the adjacent teeth against trauma compared to implants in partial and complete edentulous areas.

It is worth mentioning that different residents performed surgeries. Therefore, the operator’s skill can be considered a confounding variable. However, this is not of significant importance because surgeries were all performed by maxillofacial surgery residents under the supervision of professors. The surgeries performed by periodontal residents were excluded. Moreover, even if we classified the residents in the 5-year intervals suggested by Baqain et al,^24^ all would be in the same class of skill.

The present study had limitations because it was retrospective. These limitations include not considering the factors affecting the initial flap healing, such as flap tension, suturing quality, and complete approximation of wound margins. Therefore, it is recommended that a prospective study be conducted by considering all the confounding variables.^26,27^

## Conclusions

Despite the limitations of this retrospective study, we found significant relationships between spontaneous cover screw exposure of implants and the following factors: overlying mucosal thickness of <2 mm, fresh socket implant placement, and maxillary implant placement, especially at the location of maxillary premolars. However, further studies are necessary to confirm the present study results.

## Authors’ contributions

RN, AG, MG, and SB designed the study, AS carried out the study procedures, and all authors contributed to the manuscript writing and revising. All authors approved the final manuscript.

## Avalibility of data

The data from the reported study are available upon request from the corresponding author.

## Ethics approval

The protocol of the present study was approved by the Ethics Committee of Tabriz University of Medical Sciences under the code IR.TBZMED.REC.1399.198. All the patients signed informed consent forms.

## Competing interests

The authors declare no competing interests.
